# Influence of Climatic Factors and Nest Tree Characteristics on the Nest Structures of the Baya Weaver (*Ploceus philippinus*) in Peninsular Malaysia

**DOI:** 10.3390/ani12070815

**Published:** 2022-03-23

**Authors:** Thiruvinothini Thiruvenggadam, Marina Mohd. Top, Rosimah Nulit, Chong Leong Puan

**Affiliations:** 1 Department of Biology, Faculty of Science, Universiti Putra Malaysia (UPM), Serdang 43400, Selangor, Malaysia; toa.thiru@gmail.com (T.T.); rosimahn@upm.edu.my (R.N.); 2 Centre of Foundation Studies for Agricultural Science, Universiti Putra Malaysia (UPM), Serdang 43400, Selangor, Malaysia; 3 Faculty of Forestry and Environment, Universiti Putra Malaysia (UPM), Serdang 43400, Selangor, Malaysia; chongleong@upm.edu.my; 4 Institute of Tropical Forestry and Forest Products (INTROP), Universiti Putra Malaysia (UPM), Serdang 43400, Selangor, Malaysia

**Keywords:** baya weaver, nest, nest structure, microclimate, macroclimate

## Abstract

**Simple Summary:**

The baya weavers of South and Southeast Asia, living in colonies, build overhanging nests in partially and fully completed forms on the same trees. The female birds will only select certain nests in which to complete breeding, with nest structure linked to the nest tree characteristics and the surrounding environment. We examined nest structure, nest tree characteristics, and climatic variables for colonies found in two climatically dissimilar sites in Peninsular Malaysia. Our results indicate that nest structure is linked to nest microclimate, possibly being influenced by the surrounding temperature.

**Abstract:**

The baya weaver (*Ploceus philippinus*: Ploceidae), found across South and Southeast Asia, is known for its oblique-shaped overhanging nests. During the breeding season, females select from partially built (‘helmet’-stage) nests constructed by the males, after which nests are completed and used by the birds. Reproductive success is linked to an optimal microclimate within these nest structures. We recorded nest tree and nest structure characteristics of 66 fully completed nests for 22 colonies located in two climatically dissimilar sites in Peninsular Malaysia and examined how these factors affected the microclimate within six nests that were randomly selected at each location. Total vertical length of the nests, the height of nests from the ground, and the diameter at breast height of the nest trees recorded for the Selangor and Perlis colonies (in the southwest and north, respectively), were significantly different. The climatic variables inside and outside the nests correlated significantly in both sites. Our findings indicate that nest structures differed in Selangor and Perlis and were linked to nest microclimate, possibly being influenced by the surrounding temperature.

## 1. Introduction

Weavers (Ploceidae) are known for their elaborately woven nests that differ in size, shape, materials used, and building techniques, depending on the species. Baya weavers (*Ploceus philippinus*) live in colonies across South and Southeast Asia, and often build their nests in open habitats, including farmlands, plantations, and paddy fields, where food and nesting resources are abundant [1]. Their globular, intricately woven nests are built approximately 10 m above the ground to deter terrestrial predators [2,3].

Baya weavers use a wide selection of thin, delicate plant materials for nest-building [4] that can regulate the microclimate within a nest [5]. A microclimate is the climatic condition of a small, specialized area, a few meters or less from the ground that differs from surrounding atmospheric conditions [6]. For baya weavers, nest structure and microclimate are crucial determinants of reproductive success [7]. Different climatic conditions inside and outside the nests could be linked to the optimal internal environments required for the reproductive success [7]. For example, under high temperatures, parental regulation of the internal temperature of the nest allows sufficient aeration to increase nestling survival rates [8]. The importance of nest microclimate on reproductive success has been demonstrated in a tree cavity nester, the northern flicker (*Colaptes auratus*) of British Columbia where clutch size correlates positively with nest cavity temperature [9]. Elsewhere, experimentally increasing the temperature of tree swallow (*Tachycineta bicolor*) nests in Prince George BC, Canada, improved the growth, size, and survival rates of nestlings. [10]. As atmospheric conditions become gradually affected by climate change, the abilities of breeding birds to adjust their nest microclimates accordingly are important factors in ensuring their reproductive success [10]. The impact of microclimates on birds with enclosed nests has widely been studied in Europe among box-nesting species, such as marsh tits (*Poecile palustris*) [11], collared flycatchers (*Ficedula albicollis*) [12], and pied flycatchers (*Ficedula hypoleuca*) [13].

We measured the nest trees of the baya weaver and the structure of completed nests to determine whether the nest structure correlates with microclimatic variables at two study sites located in the northern and southwestern parts of Peninsular Malaysia. The two sites were chosen to ascertain differences in nest structure that could be linked to local climatic differences and are also where baya weaver colonies have been heavily spotted, which served as ideal locations for our study.

## 2. Materials and Methods

**Figure 1:**
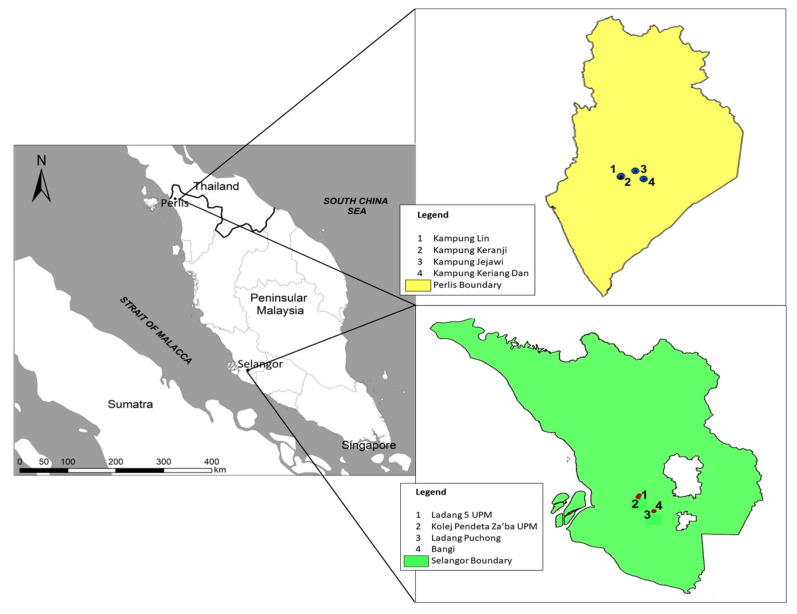
The location of the baya weaver (*Ploceus philippinus*) nest locations at the Selangor and Perlis sites.

The two study sites were located in the states of Selangor and Perlis in southwest and northeast Peninsular Malaysia, respectively, 400 km apart (Figure 1). Selangor, which is industrialized and densely populated [14], has an average annual temperature of 33 °C and precipitation of 1987 mm, while for Perlis, the average annual temperature and precipitation is 26.7 °C and 1952 mm, respectively (Malaysian Meteorological Department, 2019). Perlis receives substantial rainfall during most months of the year with a shorter dry season. The baya weaver breeds during the rainy season, which in Selangor is from late May to early October, while for Perlis, it is from November to March [1]. From August 2018 until April 2019, we measured nesting trees, nest structures, and microclimatic variables in the Selangor (3.0738 N, 101.5183 E) and Perlis (6.4449 N, 100.2048 E) sites where colonies of the bird were found in a preliminary survey of the study sites (Figure 1). These locations comprised grasslands, plantations, and rural residential areas.

**Figure 2:**
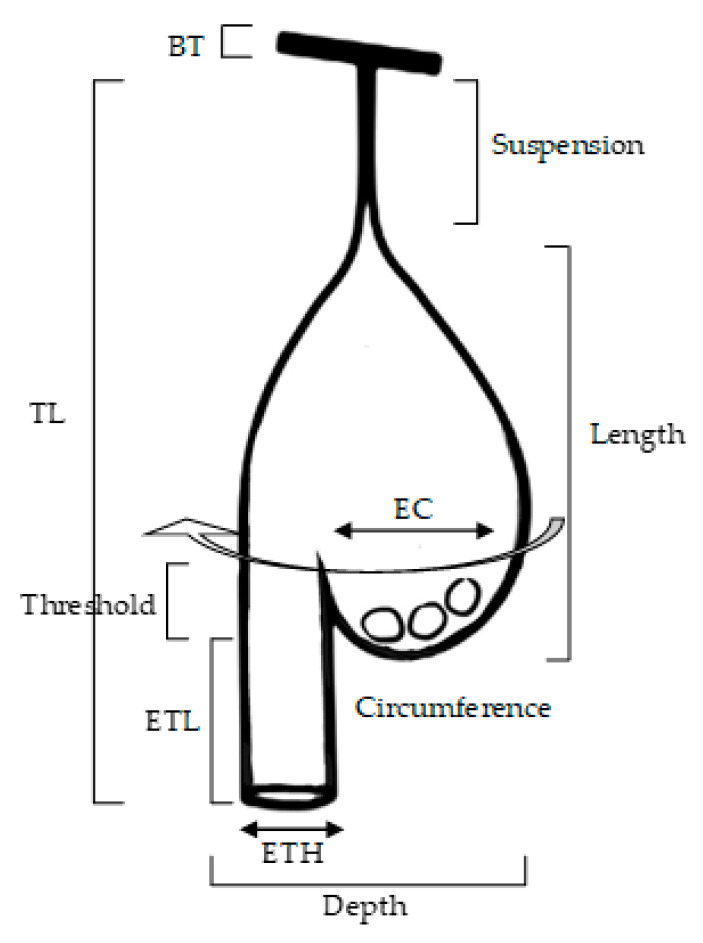
Nest structure of a complete nest of baya weaver (*Ploceus philippinus*). EC, egg chamber; ETH, entrance tube hole; BT, branch thickness; TL, total length; ETL, entrance tube length (Figure adapted from Quader [18]).

A total of 34 completed nests were assessed from 17 trees (in 12 colonies) in four locations in Selangor and 32 completed nests from 15 trees (in 10 colonies) in four locations in Perlis (Figure 1). The following measurements were recorded for completed nests: length of ‘suspension’ (from branch attachment to roof of nest), nest length (from roof to base of brood chamber), total length, depth, brood chamber diameter, entrance tube length (from base of brood chamber to rim of entrance tube), entrance tube hole, height of the threshold, which is a lip preventing eggs from falling out of the brood chamber (from top of lip to base of brood chamber), circumference, and branch thickness (Figure 2). All measurements were taken in (cm). Nest trees were chosen based on the presence of a baya weaver colony, which may comprise up to 30 nests [15]. For our study, nesting trees had at least two complete nests present. A single colony may occupy many trees [3]. For our study, nesting trees were not included in a colony if located more than 5 m from the other nesting trees in that colony. The following parameters were recorded for nesting trees: tree height (m), crown volume (cm^3^), diameter at breast height (DBH, measured using DBH tape, cm), and the height of nests from the ground (m) (Figure 2). The height of nests from the ground was also measured using a measuring tape with the help of a ladder. A handheld clinometer was used to measure tree height, which was calculated using the equation [16]:

H = (Distance from observer × tan(A)) + Base height from ground to position of clinometer

where H = Height of tree, A = Angle between the top of the tree and the ground from eye level.

The volume of spherical crown was calculated using the following formula [15]:

Spherical crown volume, V = (4/3)(π)(D/2)^3^

where D = crown diameter, taken as an average of the widest and narrowest measurement (measured using a measuring tape).

The diameter of the crown was measured by taking the sum measurement of the widest and the narrowest part of the tree crown and dividing by two. The measurements were taken using a diameter tape [17].

Macro- and microclimates in this study refer to the climatic variables outside of and within the nest, respectively. The climatic variables recorded were temperature (°C), light intensity (lux), and humidity (%RH), both inside and outside each of the 66 nests. The temperature inside the nest was measured using a laser thermometer (Model UT380, Manufacturer: OEM, China; Accuracy: ±2 °C) by pointing the laser towards the inside of the nest through the entrance hole. Light intensity and humidity were similarly measured using a 4-in-1 Lutron anemometer, hygrometer, thermometer, and light meter (Model LM-8000, Manufacturer: Lutron Electronic Enterprise, Taiwan). The atmospheric climatic measurements were also recorded using the anemometer. These measurements were taken once during the day between 9 a.m. and 12 p.m. The 24 h temperature and relative humidity of three nests from each site (six nests in total) were also recorded using a TEN1720 humidity/temperature data logger (Model TEN1720, Germany; accuracy: ±0.5 °C from −20 to 50 °C, ±1.0 °C all other ranges; ±3.0 %RH (20~80%), ±5.0 %RH (>80%)), which was mounted with flexible wires to the inner wall of the nest with care to minimize disturbance to the birds. These measurements were taken for 24 h.

To compare 24-h recording of temperature and relative humidity within the nest (microclimate) with those variables outside the nest (macroclimate), the macroclimate data were obtained from the Malaysian Meteorological Department from weather stations closest to the nesting locations in both states. The Petaling Jaya weather station was about 35 km from the Selangor site and the Kangar weather station about 3.7 km from the Perlis site.

The Mann–Whitney U test was performed using SPSS Version 23 (IBM^®^) with Bonferroni sequential correction to test whether there was any significant difference (*p* < 0.05) between macroclimate and microclimate variables. Spearman’s correlation was also performed using SPSS Version 23 (IBM^®^); it was used to examine the relationship between climatic variables for insight on significant differences between the changes in these relationships. Redundancy analysis, a linear ordination method, was performed using CANOCO 4.5 with Monte Carlo simulations to identify associations between nest structures and nest tree parameters, as well as between nest structures and microclimatic variables within the nests. F statistics was used to test the significance of each variable.

## 3. Results

Nest trees at both sites were not significantly different in height but the DBH of the Selangor trees were almost five and three times greater, respectively, than that of the Perlis trees (Table 1). Nests in Selangor were built higher off the ground than those in Perlis and this difference was significant. Regarding the nest structure, nests in Selangor were built with significantly longer vertical lengths than in Perlis.

Regarding the temperature within and outside the nests during the breeding season for each site (Table 2), the mean daily nest temperature at Selangor and Perlis was lower than the corresponding atmospheric temperature at those sites, with the Selangor nests being significantly cooler than outside temperatures. Likewise, nest humidity at Selangor and Perlis was lower than the atmospheric humidity at those sites, but the values were not significant. For Perlis, it was significantly darker inside than outside the nests, and a third lower than that in the nests in Selangor.

**Figure 3:**
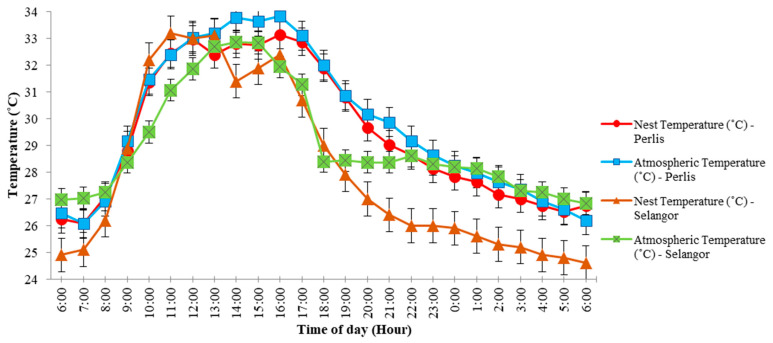
Baya weaver (*Ploceus philippinus*) nest temperature (°C) and atmospheric temperature (°C) across 34 nests in Selangor and 32 nests in Perlis, Malaysia.

**Figure 4:**
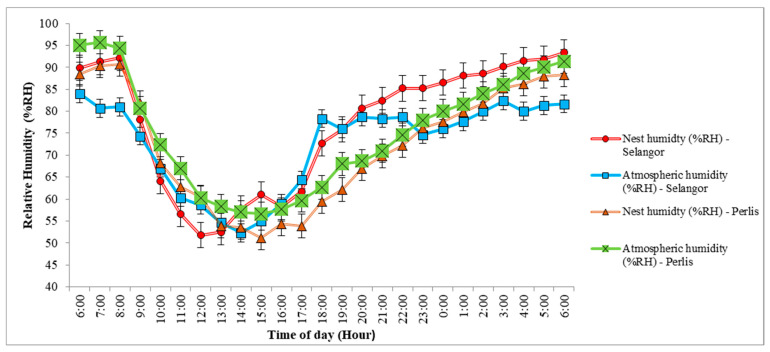
Baya weaver (*Ploceus philippinus*) nest humidity (% RH) and atmospheric humidity (%RH) across 34 nests in Selangor and 32 nests in Perlis, Malaysia.

The 24-h temperature plots within and outside the nests for both sites revealed that nest and atmospheric temperatures rose from 6:00 a.m. to peak (ca. 31–34 °C) sometime between 11:00 a.m. and 2:00 p.m., and then dropped steadily down to early morning lows (ca. 24–27 °C, Figure 3). As temperatures rose, relative humidity fell within and outside the nests at both sites, from 85–95% to lows of 50–55% between 11:00 a.m. and 2:00 p.m., and then rose to early morning maximum values (Figure 4). A closer examination of Figure 3 shows the nest temperature in Perlis was lower and fluctuated following atmospheric temperature changes while the nest temperature in Selangor fluctuated much more than the atmospheric temperature over 24 h. Nest humidity in Perlis was higher, and fluctuated following atmospheric humidity changes, while the nest humidity in Selangor showed much greater fluctuations than atmospheric humidity over 24 h (Figure 4). 

**Figure 5:**
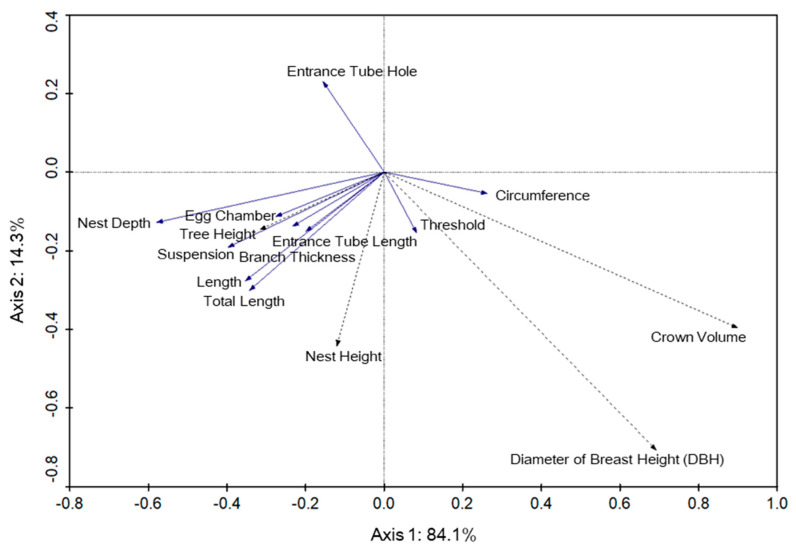
Redundancy analysis biplot of the baya weaver (*Ploceus philippinus*) nest structure (solid line vectors) and nest tree variables (dotted line vectors) for the Selangor site. Arrows indicate the direction that the nest structure increased in measurement whereas the angles between variables approximate their correlations.

The redundancy analysis using nest structure and nest tree variables indicated that in Selangor, nest structure was not significantly correlated with nest tree variable. The first and second axes explained 84.1% and 14.3% of the variance respectively, in the model (Figure 5). Species–environment correlations were 0.327 for axis 1 and 0.239 for axis 2. Species in this analysis is a reference to baya weaver nests, while environment refers to tree characteristics. The height of the nest (F = 0.14, *p* = 0.812) showed a positive association with total vertical length and the suspension of the nest, although it was not significant.

**Figure 6:**
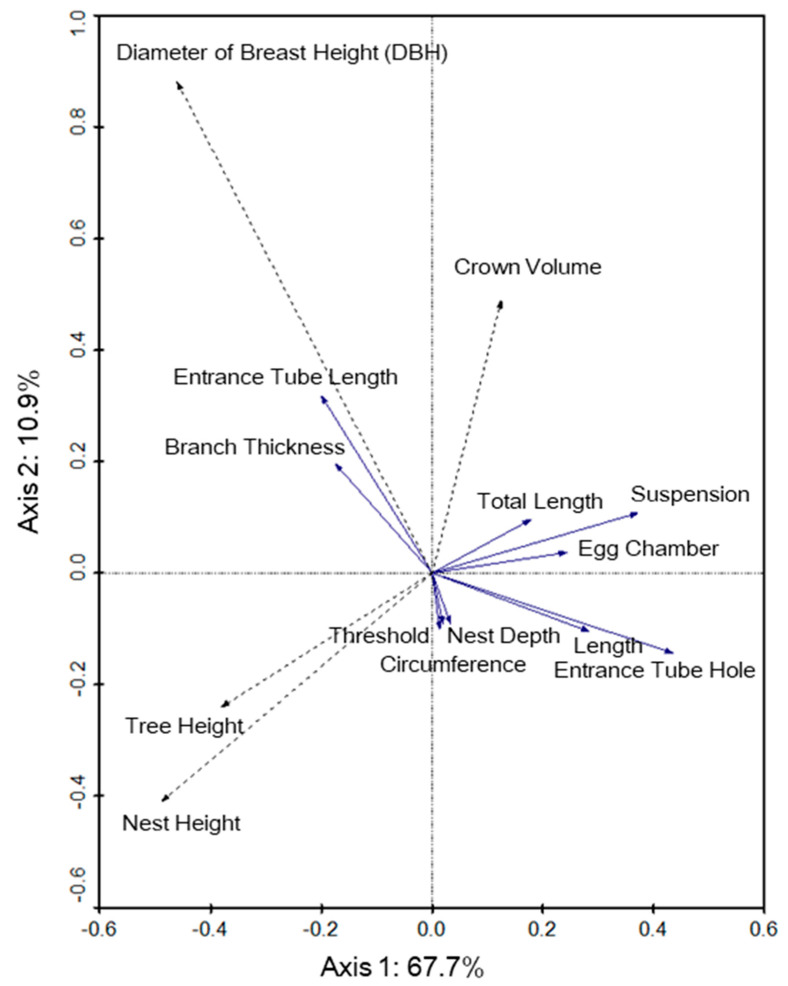
Redundancy analysis biplot of the baya weaver (*Ploceus philippinus*) nest structure (solid line vectors) and nest tree variables (dotted line vectors) for the Perlis site. Arrows indicate the direction that nest structure increased in measurement whereas the angles between variables approximate their correlations.

In Perlis, nest tree characteristics were also not significantly correlated with nest structure variables (Figure 6). The first and second axes explained 67.7% and 10.9% of the variance, respectively, in the model, and species–environment correlations were 0.330 for axis 1 and 0.408 for axis 2. Unlike Selangor, the height of the nest (F = 0.66, *p* = 0.556) showed a negative association with the total vertical length and suspension of the nest.

**Figure 7:**
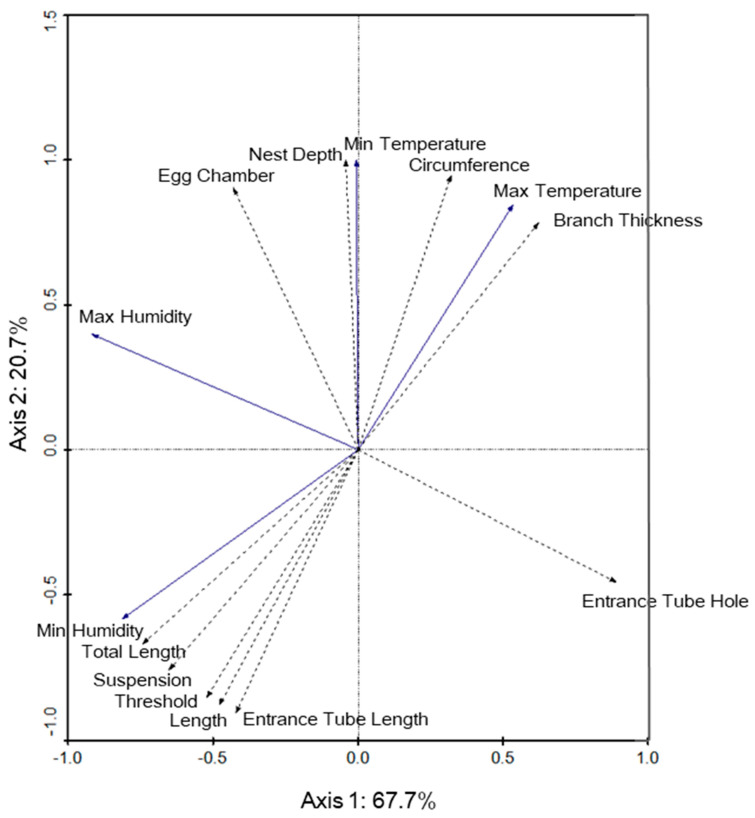
Redundancy analysis biplot of microclimate within the baya weaver (*Ploceus philippinus*) nests (solid line vectors) and nest structure variables (dotted line vectors) for the Selangor site. Arrows indicate the direction that nest structure increased in measurement, whereas the angles between variables approximate their correlations.

The redundancy analysis involving nest microclimate and nest structure variables indicated that in Selangor, the nest microclimate was not significantly correlated with nest structure. Species in this analysis is a reference to the baya weaver nest structure, while environment refers to nest microclimate. The first and second axis explained 67.7% and 20.7% of the variance, respectively, in the model and species–environment correlations, were 0.330 for axis 1 and 0.408 for axis 2 (Figure 7). Two nest structures, i.e., entrance tube hole (F = 1.79, *p* = 0.168) and length (F = 0.00, *p* = 1.000) linked to microclimate were notable, although the correlations were not significant.

**Figure 8:**
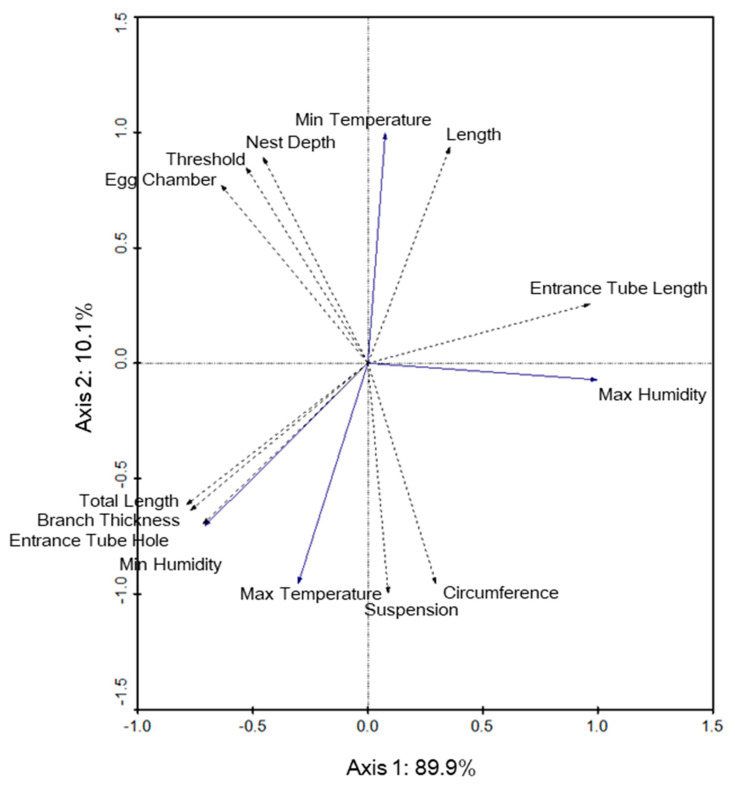
Redundancy analysis biplot of microclimate within the baya weaver (*Ploceus philippinus*) nests (solid line vectors) and nest structure variables (dotted line vectors) for the Perlis site. The arrows indicate the direction that the nest structure increased in measurement whereas the angles between variables approximate their correlations.

Likewise, in Perlis, nest microclimate was not significantly correlated with nest structure. The first and second axes explained 89.9% and 10.1% of the variance, respectively, in the model, and species–environment correlations were 1.00 for axis 1 and 1.00 for axis 2 (Figure 8). Two nest structures linked to microclimate were notable, although the correlations were not significant, i.e., for the entrance tube hole (F = 5.51, *p* = 0.344) and length (F = 0.00, *p* = 1.000).

Spearman’s correlation analysis performed between macroclimate and microclimate factors at Selangor and Perlis (Table 3 and Table 4) showed that atmospheric light intensity had a significant negative correlation with the temperature inside the nests. Interestingly, there was no significant correlation between atmospheric and nest light intensity in Selangor, but a significant negative correlation was observed in Perlis. A negative relationship was also seen in Selangor, albeit not significant. In Selangor, the atmospheric temperature showed a significant negative correlation with the nest’s relative humidity.

## 4. Discussion

The significantly higher nests in Selangor than those found in Perlis could be due to a higher predator threat in Selangor––weavers are reported to build their nests higher off the ground if there is a high predation risk [19]. In India, two studies reported the height of nests to be in the range of 2.0–5.3 and 2.15–3.69 m, respectively [3,20]. These ranges are close to what we found. However, some nests were built lower than 2 m above the ground, which may indicate that the predatory risk in Malaysia is lower than in India. Further studies would be necessary to untangle the predation risk in our study area. The significantly shorter vertical lengths of nests in Perlis colonies as compared with those in Selangor could be attributed to several possible reasons, such as nest tree size and weather-related hazards [3]. Trees in the northern states, such as Perlis, suffer more wind-related damage than in southern states, such as Selangor [21]. Hence, baya weavers in Perlis may build their nests with shorter lengths to accommodate stronger winds. The larger trees with bigger canopies (as evinced by their significantly greater DBH) colonized by the birds in Selangor may have provided a better buffer against the elements than the smaller trees in Perlis; thus, allowing for more elongated nests to be built higher off the ground in Selangor. The trade-off faced by the Perlis colonies with nests closer to the ground may be a higher predation risk, which was not assessed in our study. Based on our observation, nests that were built very low on trees in Perlis often can be seen overhanging above water bodies, which is a strategy to deter predators, and has been recorded in previous studies [18,19]. This observation can be seen in Figure S1.

The changes in climatic factors inside the nest were influenced by the changes in atmospheric climatic factors [7,22]. Thus, in reference to Table S1, it can be inferred that the higher atmospheric temperature in Selangor than Perlis necessitated the construction of nests in Selangor that provide lower nest temperatures. Some previous studies indicated that the microclimate inside the nest is important for birds regardless of whether they are in embryonic, nestling, or adult stages [8,23]. In Selangor, the temperature inside and outside the nests were found to be significantly different (*p* < 0.05), while in Perlis, the light intensity inside and outside the nests were significantly different (*p* < 0.05). It is important for birds to regulate the temperature inside the nest for incubation, as extreme temperatures can be fatal for parents and offspring alike [22,24]. In reference to Table 2, temperatures inside the nest were often lower than outside. This corroborated with a study in India [4] that recorded a similar pattern in in temperature of baya weaver nests. Such temperature differences may be important for the birds in terms of maintaining essential activities, such as embryo developments that enhance survival rates in harsh climates, as proven in previous studies by experimental manipulation [22]. However, further study is required to investigate the effects of temperature differences in baya weaver nests on fitness and survival of the chicks. The nest of the baya weaver, being a closed structure, leads to a lower light intensity inside the nest, regulated by the fibers used to weave the nest that create pores, allowing minimal light to enter the nest [7].

The significant negative correlation between atmospheric light intensity and the temperature inside the nests in Selangor and Perlis implies that the impact of high atmospheric light intensity may have been regulated by the birds through creating little pores in the nest wall, allowing limited light penetration into the nest [7]. As a result, this may prevent the nest temperature from increasing, which can be otherwise detrimental to the health of the offspring [4,25]. Previous studies have demonstrated that the type of nest materials used may influence heat regulation in the nest where the materials used in the outer part of the nest were thicker compared to the inner part of the nest [26]. The amount of air trapped in the nest wall could affect nest insulation. Thus, it can be inferred that the nest wall could play a role in increasing the rate of heat loss, decreasing the temperature of the nest. However, more in depth study is required to investigate this matter, as the type of nest materials used could vary between nests of different colonies. The significant negative correlation between atmospheric and nest light intensity for nests in Perlis indicates that this regulation may be important to prevent the nest from overheating. A previous study has proven that nests exposed to a high level of sunlight increases the temperature of the nests [27]. Another study, featuring the southern yellow-billed hornbill (*Tockus leucomelas*) in arid areas with high temperatures (32.3 to 39.1 °C), provided insight into how fledgling success was dramatically reduced with temperatures above 35.1 °C [26]. In this study, the maximum temperatures of baya weaver nests were 37.90 °C and 37.80 °C in Selangor and Perlis, respectively, as described in Table S1. Further studies are recommended to expand on the effects of high temperatures on the fledgling success rate of baya weavers. The significant negative correlation between atmospheric temperature and nest humidity in Selangor sites could mean that an increase in air temperature is expected to lower relative humidity in the air [28,29] which in turn decreases relative humidity inside the nest [30].

The results of redundancy analyses of nest structure measurements against nest tree variables in Selangor indicate that the increase of nest height will likely lead to an increase in total vertical length of the nests. This was the opposite in Perlis, where the increase of nest height will likely lead to a decrease in the total vertical length of the nest. Nests built at high heights from the ground have been proven to experience reduced risks of predation and increased nesting success [19,31]. Hence, it can be inferred that tall trees may be safer for the nest, enabling baya weavers to build long nests, while nests built on short trees are forced to have shorter structures that increase the risk of predation. The difference shown in Perlis could be caused by wind factors, which, as discussed previously, affect the nest structure.

On the other hand, the maximum temperature was negatively correlated with entrance tube length (ETL) and nest length in both Selangor and Perlis. It is possible that a trade-off relationship [32] involving the temperature within the nest and nest structure may exist, i.e., the nest structure shape may be compromised to maintain nest insulation and reduce overheating of the nest, especially when the temperature is high. This hypothesis was previously tested for some birds that seem tolerant to trade-offs between nest insulation quality and nest structure at the expense of the nest being more exposed to predators [5,32].

It is unclear how one or all nest structure characteristics can alter the temperature inside the nest, since some structures may not contribute toward regulating the climatic conditions inside the nest, e.g., the suspension that supports the body of the nest, the entrance tube that acts as a doorway for the birds, the branch diameter that supports the nest in strong winds, and the threshold that prevents eggs from falling out of the nest during strong winds [30]. Previous studies have also demonstrated that the nest temperature is influenced by the type of fibers used to build the nest—that act to cool it [7]. Similarly, some birds in other habitat types may use mud inside the nest as a form of insulation [33]. Baya weavers are known to line the interior walls of the nest with mud to fortify the nest against strong winds, repel rain, and possibly keep the nest cool, internally, through the insulation properties of the mud [34,35]. It can be concluded that while the nest structure may have a trade-off relationship with nest microclimate, the nest structure may not affect the nest microclimate directly. Hence, further study is required to investigate the type of nest materials used by baya weavers and the insulation properties of these materials as well as the changes in the microclimate, if any, following different nest stages and the nesting success of the birds. The baya weaver is currently classified as a species of ‘least concern’ on the IUCN Red List, nevertheless a better understanding of how rapid urbanization and climate change may affect its nesting ecology and population is beneficial. This study adds to the baseline information on baya weaver nesting ecology. Understanding the factors influencing the nesting ecology of baya weavers will help with future targeted conservation interventions, for this and other tropical bird species that build overhanging nests.

## 5. Conclusions

This study found that the nest structure influences the microclimate of the nest, in such a manner that differs significantly from the macroclimate that surrounds the nest of baya weavers. Several nest structures in Selangor and Perlis, such as entrance tube length, length, or total vertical length, correlated negatively with microclimate factors, such as temperature. The association between nest structure and nest temperature would indicate that some properties of the nest structure of the baya weaver show thermoregulatory properties, isolating the inside of the nest from the surrounding environment. This information can pave a way towards future studies where the relationship between climatic factors and nest building of baya weavers may be studied further.

## Figures and Tables

**Table 1:** Characteristics of the baya weaver (*Ploceus philippinus*) nest trees and active nests at the Selangor and Perlis colonies. Values are average ± SE, with n in parentheses. *p*-values based on Mann–Whitney U tests.

Parameters	Selangor	Perlis	Mann–Whitney U	*p*-Value	Bonferroni Adjusted *p*-Value
Tree height (m)	6.4 ± 3.32 (17)	6.4 ± 1.33 (15)	117.00	0.710	0.010
Crown volume (cm^3^)	429.1 ± 573.26 (17)	89.7 ± 106.55 (15)	72.50	0.037	0.005
DBH (cm)	58.8 ± 16.96 (17)	18.3 ± 3.66 (15)	52.00	0.003	0.004 *
Branch thickness (cm)	3.2 ± 0.96 (34)	3.8 ± 0.77 (32)	358.00	0.017	0.004
Height of nest (m)	3.4 ± 0.73 (34)	2.8 ± 0.55 (32)	229.00	0.001	0.003 *
Total vertical length (cm)	65.2 ± 16.93 (34)	54.9 ± 16.28 (32)	315.50	0.001	0.002 *
Nest length (cm)	63.2 ± 19.54 (34)	40.9 ± 13.69 (32)	403.00	0.070	0.007
Nest depth (cm)	18.3 ± 4.34 (34)	18.7 ± 2.36 (32)	515.00	0.710	0.012
Suspension (cm)	30.1 ± 13.28 (34)	23.7 ± 12.68 (32)	366.00	0.022	0.005
Circumference (cm)	43.4 ± 9.77 (34)	43.4 ± 4.66 (32)	524.50	0.802	0.016
Egg chamber (cm)	10.2 ± 1.94 (34)	10.5 ±2.23 (32)	534.00	0.898	0.050
Entrance tube hole (cm)	8 ± 1.76 (34)	9.5 ±8.80 (32)	526.00	0.067	0.006
Entrance tube length (cm)	17.8 ± 8.25 (34)	13.7 ± 5.01 (32)	401.50	0.817	0.025
Threshold (cm)	10.1 ± 12.98 (34)	7.3 ± 1.59 (32)	435.00	0.161	0.008

Note: Values noted with * are Bonferroni-corrected significant values.

**Table 2:** Mann–Whitney U tests correlating macroclimate and microclimate variables of active nests of baya weavers (*Ploceus philippinus*) in Selangor and Perlis (Malaysia).

	Atmospheric	Nest	Mann–Whitney U	*p*-Value	Bonferroni Adjusted *p*-Value
Selangor (*n* = 34)					
Temperature (°C)	33.8 ± 2.4	30.7 ± 3.1	83.500	<0.001	0.016 *
Light intensity (lux)	11,503 ± 5745	2099 ± 5373	408.500	0.05	0.025
Humidity (%RH)	54.9 ± 9.7	54.3 ± 8.1	568.000	0.849	0.05
Perlis (*n* = 32)					
Temperature (°C)	34.1 ± 2.7	32.4 ± 2.7	468.500	0.559	0.05
Light intensity (lux)	12,765 ± 5248	738 ± 1227	6.000	<0.001	0.016 *
Humidity (%RH)	64.1 ± 9.31	57.2 ± 12.4	361.000	0.026	0.025

Note: Values noted with * are Bonferroni-corrected significant values.

**Table 3:** Strength of correlations and their significance levels based on Spearman’s correlation analysis between macroclimate and microclimate factors at Selangor site.

**Microclimate Factors**		**Macroclimate Factors**		
	**Spearman’s Rho**	**Temperature (°C)**	**Light Intensity (lux)**	**Humidity (%RH)**
	Temperature (°C)	0.127	−0.481 **	0.014
	Light intensity (lux)	−0.058	−0.79	0.336
	Humidity (%RH)	−0.625 **	0.306	0.613 *

Note: **, correlation is significant at the level 0.01 (two-tailed); *, correlation is significant at the level 0.05 (two-tailed).

**Table 4:** Strength of correlations and their significance levels based on Spearman’s correlation analysis between macroclimate and microclimate factors at Perlis site.

**Microclimate Factors**		**Macroclimate Factors**		
	**Spearman’s Rho**	**Temperature (°C)**	**Light Intensity (lux)**	**Humidity (%RH)**
	Temperature (°C)	0.539 **	−0.652 **	−0.131
	Light intensity (lux)	0.042	−0.357 *	0.285
	Humidity (%RH)	−0.036	−0.268	0.359 *

Note: **, correlation is significant at the level 0.01 (two-tailed); *, correlation is significant at the level 0.05 (two-tailed).

## Data Availability

Data sharing is not applicable to this article.

## Supplementary Materials

The following supporting information can be downloaded at: https://www.mdpi.com/article/10.3390/ani12070815/s1, Figure S1: Nests of baya weavers (*Ploceus philippinus*) above water body in Perlis; Table S1: Descriptive statistics of macroclimates and microclimate factors of baya weaver (*Ploceus philippinus*) nests.
