# Supplementary material for: Influence of Climatic Factors and Nest Tree Characteristics on the Nest Structures of the Baya Weaver (Ploceus philippinus) in Peninsular Malaysia
Source: Animals (Basel). 2022 Mar 23;12(7):815. doi: 10.3390/ani12070815 (PMC8996917; doi:10.3390/ani12070815)
Supplement: Supplementary file 1 [file animals-12-00815-s001.zip › Supplementary Materials.pdf]

Table S1: Descriptive statistics of macroclimates and microclimate factors of Baya Weavers (*Ploceus philippinus*) nests

	State	Number of nests (n)	Range	Mean ± Standard deviation
**Macroclimate factors**				
**Temperature (°C)**	Selangor	34	29.40 – 37.90	33.84 ± 2.38
	Perlis	32	29.20 – 37.80	34.15 ± 2.65
**Humidity (%RH)**	Selangor	34	40.30 – 85.00	54.97 ± 9.68
	Perlis	32	37.90 – 76.40	64.11 ± 9.31
**Light intensity (lux)**	Selangor	34	2036.00 – 19770.00	11503.06 ± 5745.29
	Perlis	32	843.00 – 25015.00	12765.00 ± 5248.41
**Microclimate factors**				
**Temperature (°C)**	Selangor	34	25.10 – 37.40	30.712 ± 3.09
	Perlis	32	28.20 – 37.90	32.44 ± 2.75
**Humidity (%RH)**	Selangor	34	35.30 – 68.20	54.27 ± 8.07
	Perlis	32	29.60 – 77.80	57.16 ± 12.37
**Light intensity (lux)**	Selangor	34	4.00 – 3168.00	2099.71 ± 5373.47
	Perlis	32	18.00 – 4160.00	738.16 ± 1227.23

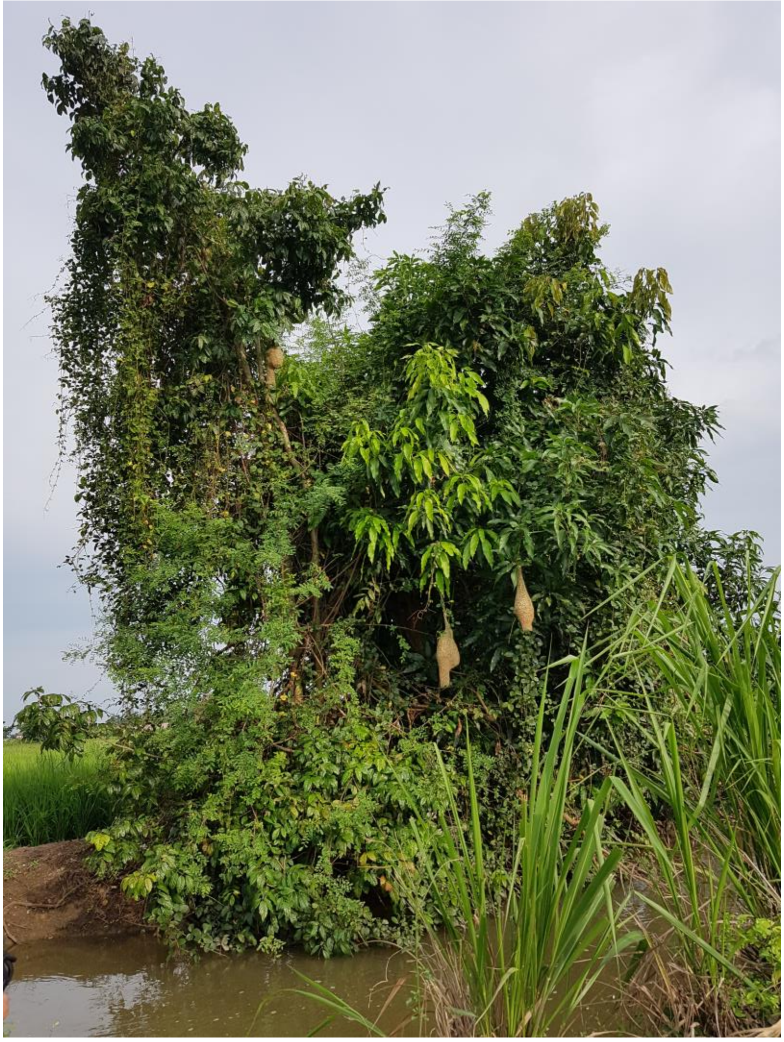

Figure S1: Nests of Baya Weavers (*Ploceus philippinus*) above water body in Perlis
