# Supplementary figures and images for: Influence of Climatic Factors and Nest Tree Characteristics on the Nest Structures of the Baya Weaver (Ploceus philippinus) in Peninsular Malaysia
Source: Animals (Basel). 2022 Mar 23;12(7):815. doi: 10.3390/ani12070815 (PMC8996917; doi:10.3390/ani12070815)

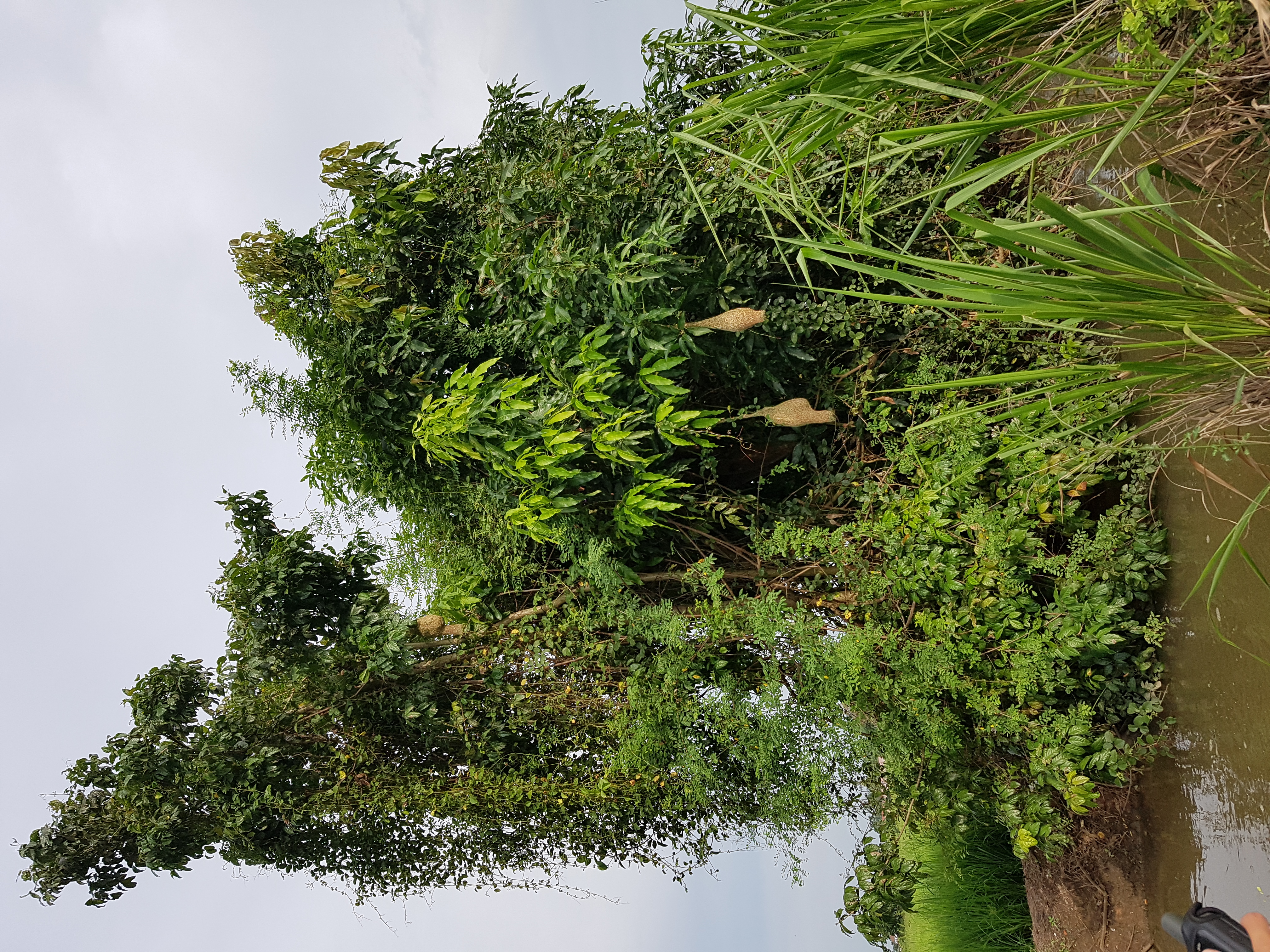

Supplement: Supplementary file 1 [file animals-12-00815-s001.zip › Figure S1.jpg]
